# A Straightforward Hypoxic Cell Culture Method Suitable for Standard Incubators

**DOI:** 10.3390/mps4020025

**Published:** 2021-04-08

**Authors:** Svea Matthiesen, Rico Jahnke, Michael R. Knittler

**Affiliations:** Institute of Immunology, Friedrich-Loeffler-Institut (FLI), 17493 Greifswald-Insel Riems, Germany; svea.matthiesen@fli.de (S.M.); rico.jahnke@fli.de (R.J.)

**Keywords:** hypoxia, normoxia, cell culture, standard incubator

## Abstract

We present a new and straightforward method by which standard cell culture plates can be sealed off from ambient air and be placed under controlled hypoxic cell culture conditions without costly or highly specialized materials. The method was established on a murine cell culture system using the dendritic cell line JAWS II but can be readily adapted to other cell cultures. The procedure was designed to be easy to implement in cell culture laboratories with standard incubators and requires only readily available materials, resources, and consumables, such as six-well plates, degassed culture medium, CoCl_2_, a vacuum sealer, etc., and no further complicated laboratory equipment. The simple hypoxic cell culture method presented here is technically reliable and experimentally safe. As it can be performed in any standard incubator, it is suitable for use at both low and higher biosafety levels.

## 1. Introduction

The oxygen concentration of normal ambient air is 21% (paO_2_ = 160 mmHg). However, it is well known that the partial pressure of oxygen in the blood decreases significantly from the arteries to the cells in the tissues [1]. Specifically, while a paO_2_ of 80–100 mmHg is found in the pulmonary vessels, it decreases to 40–60 mmHg in the arterioles and is only between 1 and 20 mmHg (0.1–3%) in the cells. [1]. O_2_ serves the mitochondria as a terminal electron acceptor for energy production, and one important question in biological research is how cells adapt to low O_2_ concentrations (hypoxia), which can develop in cells/organs during inflammation [2,3] (e.g., after viral or bacterial infections [4,5]), cancer, and metabolic diseases [6,7]. As a specific response to hypoxia, transcriptional upregulation of specific cellular genes necessary to maintain cellular homeostasis and survival occurs [8]. Thus, glycolysis is strongly induced in order to facilitate anaerobic ATP synthesis by the cell [9]. Hypoxia-inducible factor-1 (HIF-1: consisting of α and β subunits) plays a crucial role in this process as a master regulator [10]. When cellular hypoxia occurs (≤2%), HIF-1 is immediately available to the hypoxic cells (within seconds) and enables their survival [11]. However, in the field of infection immunology, research on host–pathogen interaction in the context of hypoxia is still lacking, but it could be essential for developing future therapeutic strategies. To study hypoxia in cell culture, sophisticated hypoxia incubators are usually used to create low-oxygen hypoxic atmospheres; however, this approach is very investment/cost intensive. For initial pilot experiments and/or research projects that do not focus exclusively on hypoxia, such equipment expenses can be prohibitive. Moreover, at higher biocontainment levels (BSL3, and 4), a simple technical solution for hypoxia studies that allows not only safe and easy handling along with appropriate disposal possibilities represents an important experimental advantage. 

## 2. Experimental Design

**Figure 1:**
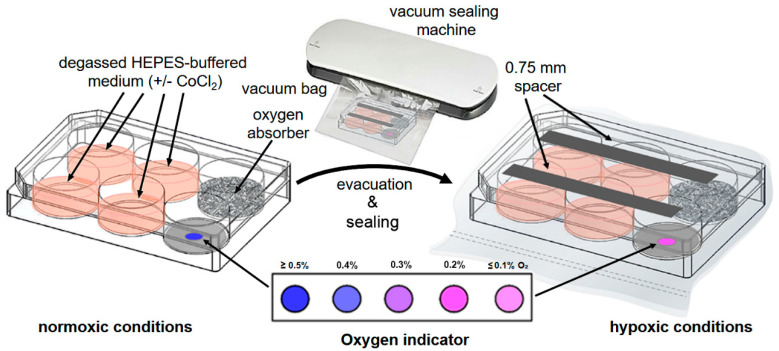
Sealed hypoxic cell culture plate. For hypoxic cell culture conditions, JAWS II cells are seeded under normoxic conditions in degassed, HEPES-buffered complete IMDM (selected wells are additionally supplemented with CoCl_2_). Moreover, an oxygen absorber and an oxygen sensor (AgelessEye) are added to the 6-well plate to establish and monitor the low oxygen environment, respectively. Two sterile 0.75 mm plastic spacers placed on top of the 6-well plate allow gas exchange between the different wells of the cell culture plate. After the cell culture plate is closed with the lid, it is placed in a vacuum bag, evacuated from ambient air, and sealed using a vacuum sealing machine. Finally, the sealed hypoxic cell culture plate is then placed in a standard incubator at 37 °C. When exposed to low oxygen (≤0.5% O_2_ concentration), the oxygen sensor AgelessEye turns from blue to pink. This color change can be controlled/monitored throughout the cultivation of the cells.

This article describes a methodical procedure (Figure 1) in which cell culture plates sealed from ambient air can be put under hypoxic conditions without significant equipment and material costs. The cell culture vessel consists of a standard 6-well cell culture plate, in which one of the six available wells contains 0.3 g of oxygen absorber [12] (a defined mixture of iron powder (≤60% (*w/w*)), sodium salts (≤20% (*w/w*)), and activated carbon (≤20% (*w/w*)) [13], which is according to manufacturer able to sustainably reduce O_2_ in the entire volume of the 6-well plate (100 cm^3^). Another well of the plate contains a reversible resazurin/resorufin-based oxygen indicator [14] (e.g., AgelessEye), which monitors the current oxygen content (in the range ≤ 0.1%–≥0.5%) within the 6-well plate. When exposed to oxygen, the AgelessEye turns from pink to purple to blue then returns to its original pink color as the oxygen in the cell culture plate is reduced. The remaining four wells of the culture plate can be utilized for different cell culture approaches. Degassed/HEPES-buffered cell culture medium reduces the oxygen tension before the actual hypoxia experiment starts. For practical use, the 6-well plate is also evacuated in suitable shrink-wrap by a vacuum sealer machine, and thus a large part of the atmospheric O_2_ is already reduced. Sterile “plastic spacers” (placed between the lid and the 6-well plate) guarantee that the entire cell culture vessel with all six round chambers has a uniform hypoxic atmosphere. To maintain the hypoxic cellular state when opening the 6-well plate after culturing, 100 µM CoCl_2_ is added preventively to the medium and all other solutions and buffers. This stabilizes the cellular hypoxia regulator HIF-1α and the cellular downstream processes controlled by it [15]. The cell cultures’ hypoxic status can be directly monitored by AgelessEye, fluorescent hypoxia live cell dye, increased glycolytic lactate production or HIF-1α stabilization, and pyruvate dehydrogenase kinase 1 (PDK1) induction. The advantages of this new simple method are as follows: fast and stable generation of a hypoxic cell culture environment, availability of required material, simple and safe disposal of the device after the entire experiment is finished, as well as cheapness along with relatively low time costs for preparation of the sealed hypoxic cell culture plate.

### 2.1. Materials

- 1.5 mL Safe-Lock tubes (Eppendorf, Hamburg, Germany, Cat. No.: 0030120086);
- 15 mL conical tubes (Greiner Bio-One, Frickenhausen, Germany; Cat. No.: 10384601);
- 2-Mercaptoethanol (Roth, Karlsruhe, Germany, Cat. No.: 4227.2);
- 50 mL conical tubes (Greiner Bio-One, Frickenhausen, Germany; Cat. No.: 10711212);
- 6-well plate (Greiner Bio-One, Frickenhausen, Germany; Cat. No.: 657160);
- Acrylamide/bis—Rotiphorese Gel 30 (Roth, Karlsruhe, Germany, Cat. No.: 3029.2);
- AgelessEye (oxygen indicator) (Mitsubishi Gas Chemical Company, Tokyo, Japan; Distributor: Long Life for Art, Cat. No.: O2INDAEYE);
- Amphotericin B (Thermo Fisher Scientific, Schwerte, Germany, Cat. No.: 15290018);
- Anti-GAPDH antibody (Merk, Darmstadt, Germany, Cat. No.: CB1001-500UG);
- Anti-HIF-1α antibody (Novus Biologicals, Centennial, USA, Distributor: Bio-Techne GmbH, Wiesbaden, Germany Cat. No.: NB100-479);
- Anti-mouse IgG-HRP (Jackson ImmunoResearch, West Grove, U.S.A., Distributor: Dianova, Hamburg, Germany, Cat. No.: 115-035-146);
- Anti-rabbit IgG-HRP (Jackson ImmunoResearch, West Grove, U.S.A., Distributor: Dianova, Hamburg, Germany, Cat. No.: 111-035-144);
- Anti-PDK1 antibody (Novus Biologicals, Centennial, USA, Distributor: Bio-Techne GmbH, Wiesbaden, Germany, Cat. No.: NB100-2384SS);
- APS (Ammonium peroxodisulfate) (Roth, Karlsruhe, Germany, Cat. No.: 9178.2);
- Blotting filter paper MN 218 B (Macherey-Nagel, Düren, Germany, Cat. No.: 742113);
- Bromophenol blue (Sigma-Aldrich, Munich, Germany, Cat. No.: B0126-25G);
- CoCl_2_ (cobalt chloride) Merk, Darmstadt, Germany; Cat. No.: 232696-5G);
- FCS (fetal calf serum), 0.2 µm sterile filtered (Pan Biotech, Aidenbach, Germany, Cat. No.: P30-3302);
- Formaldehyde, 37% (Roth, Karlsruhe, Germany, Cat. No.: 6967.2);
- Glycerin (Roth, Karlsruhe, Germany, Cat. No.: 6967.2);
- Glycine (Roth, Karlsruhe, Germany, Cat. No.: T873.2);
- HCl (hydrogen chloride), 37% (Roth, Karlsruhe, Germany, Cat. No.: 4625.1);
- HEPES (4-(2-hydroxyethyl)-1-piperazineethanesulfonic acid) (Serva, Heidelberg, Germany; Cat. No.: 25245.02);
- Image-iT Green Hypoxia Reagent (Fisher Scientific, Schwerte, Germany, Cat. No.: 15940773);
- IMDM (Iscove’s Modified Dulbecco’s) Medium (Fisher Scientific, Schwerte, Germany; Cat. No.: GibcoTM21980065);
- JAWS II (American Type Culture Collection (ATCC), Manassas, U.S.A., Cat. No.: CRL-11904);
- KCl (potassium chloride) (Roth, Karlsruhe, Germany, Cat. No.: 6781.1);
- KH_2_PO_4_ (potassium hydrogen phosphate) (Roth, Karlsruhe, Germany, Cat. No.: 3904.1);
- L-lactate-assay-kit (Sigma-Aldrich, Cat. No.: MAK329-1KT);
- MINI-Vertical Spacer, 0.75 mm (Roth, Karlsruhe, Germany Cat. No.: N624.1);
- Mouse GM-CSF (granulocyte-macrophage colony-stimulating factor), recombinant protein (Thermo Fisher Scientific, Schwerte, Germany, Cat. No.: PMC2011);
- Na_2_HPO_4_ (sodium hydrogen phosphate) (Roth, Karlsruhe, Germany, Cat. No.: 4984.1);
- NaCl (sodium chloride) (Roth, Karlsruhe, Germany, Cat. No.: 3957.1);
- Nitrocellulose membrane, 0.45µm (Hartenstein, Würzburg, Germany, Cat. No.: 10600002);
- Oxygen absorber (O2frepak, Guangdong, China; Distributor: Amazon, Germany, Cat. No.: O2frepak 100CC);
- Penicillin–streptomycin (5000 U/mL) (Thermo Fisher Scientific, Schwerte, Germany, Cat. No.: 15070063);
- Pierce ECL Western Blotting Substrate (Thermo Fisher Scientific, Schwerte, Germany, Cat. No.: 32209);
- Pipette Tips, 1–200 μL (Thermo Fisher Scientific, Schwerte, Germany, Cat. No.: 11923446);
- Pipette Tips, 200–1000 μL (Greiner Bio-One, Frickenhausen, Germany, Cat. No.: 10557071);
- Polyoxyethylene (20)-sorbitan monolaurate (Tween 20) (VWR, Darmstadt, Germany, Cat. No.: M147-1L);
- Ponceau S (Roth, Karlsruhe, Germany, Cat. No.: 5938.2);
- Prestained protein ladder (Thermo Fisher Scientific, Schwerte, Germany Cat. No.: 26616);
- ProLong^™^ Gold antifade reagent with DAPI (mounting medium) (Thermo Fisher Scientific/Invitrogen, Schwerte, Germany, Cat. No.: P36931);
- Protease inhibitor (cOmplete ULTRA Tablets, EDTA-free) (Roche, Basel, Switzerland, Distributor: Sigma-Aldrich, Munich, Germany, Cat. No.: 5892953001);
- Scalpel (Roth Karlsruhe, Germany, Cat. No.: X004.1);
- SDS (sodium dodecyl sulfate) (Roth, Karlsruhe, Germany, Cat. No.: 2326.2);
- Skim milk powder (Heirler-Cenovis, Radolfzell, Germany, Cat. No.: 4010318030305);
- TEMED (N, N, N′, N′- tetramethylethylene-diamine) (Roth, Karlsruhe, Germany, Cat. No.: 2367.3);
- Transparent foil (Roth Karlsruhe, Germany, Cat. No.: 1255.1);
- Trichloroacetic acid (TCA)-Deproteinization Kit—Deproteinizing Sample Preparation Kit II (Sigma-Aldrich, Munich, Germany, Cat. No.: MAK342-1KT);
- Tris (Thermo Fisher Scientific, Schwerte, Germany, Cat. No.: 15504020);
- Trypan blue (Roth, Karlsruhe, Germany, Cat. No.: CN76.2);
- Trypsin-EDTA (Ethylenediaminetetraacetic acid) solution (Sigma-Aldrich, Munich, Germany, Cat. No.: E8008-100ML);
- Urea (Roth Karlsruhe, Germany, Cat. No.: 7638.1);
- Vacuum bags (Shenzhen Green Electrical Appliance Co., Ltd, Guangdong, China; Distributor: Amazon; Cat. No.: 11 × 16/100);
- X-ray film, UV/blue sensitive Super RX-N (Fujifilm Europe GmbH, Düsseldorf, Germany, Distributor Hartenstein, Würzburg, Germany, Cat. No.: RF11).

### 2.2. Equipment

- Bio-Rad Trans-Blot Cell (Bio-Rad Laboratories GmbH, Feldkirchen, Germany, Cat. No.: 1703930);
- Biological safety cabinet—Berner Flow Safe (Berner, Elmshorn, Germany, Cat. No.: B-[MaxPro]^2^-130);
- Centrifuge—Eppendorf 5420 (Eppendorf, Hamburg, Germany, Cat. No.: 5420000318);
- Centrifuge—Hettich Rotina 380R (Hettich, Vlotho, Germany, Cat. No.: 1706);
- Digital camera—Panasonic DMC-TZ18 Lumix (Panasonic Germany, Wiesbaden, Germany, Cat. No.: EAN 5025232608850);
- ELISA Reader—Sunrise Remote (Tecan, Gröding, Austria, Cat. No.: F039300);
- Film developer—Compact 2 (PROTEC GmbH & Co. KG, Oberstenfeld, Germany, Cat. No.: 11951-1111-5810);
- Fluorescence microscope—Zeiss Axiovert 200 with ApoTome unit (Carl Zeiss Light Microscopy, Göttingen, Germany, Cat. No.: Axiovert 200 including ApoTome 423660-0000-00);
- Heating block—Thermomixer comfort 5355 (Eppendorf, Hamburg, Germany Cat. No.: 022670107);
- Hypoxia Cabin—Whitley H35 Hypoxystation (Don Whitley Scientific Pty Ltd, Bingley, GB, Cat. No.: MEA06060);
- Incubator—Sanyo MCO-19AIC CO_2_ (Sanyo E & E, Europe B.V., The Netherlands, Cat. No.: 5519188);
- Inverted microscope—ECLIPSE TS100-F (Nikon, Düsseldorf, Germany, Cat. No.: *TS100*-*F*);
- Microscope camera—DFK 21AU04.AS (The Imaging Source Europe GmbH, Bremen, Germany, Cat. No.: DFK 41AU02);
- Microscope LED illumination source—CoolLED pe-200 (CoolLED, Andover, G.B., Cat. No.: CoolLED pE-200);
- Neubauer counting chamber (Roth, Karlsruhe, Germany, Cat. No.: T728.1);
- pH meter—Hanna Checker (HANNA Instruments, Vöhringen, Germany, Cat. No.: Z35109);
- Pipette 100–1000 µL (Eppendorf, Hamburg, Germany, Cat. No.: 3123000063);
- Pipette 20–200 µL (Eppendorf, Hamburg, Germany, Cat. No.: 3123000055);
- Pipette 2–20 µL (Eppendorf, Hamburg, Germany, Cat. No.: 3123000098);
- Power supply—Bio-Rad PowerPac Universal Power Supply (Bio-Rad Laboratories GmbH, Feldkirchen, Germany Cat. No.: 1645070);
- Rocking platform—Heidolph Duomax 1030 (Heidolph Instruments GmbH & CO. KG, Schwabach, Germany, Cat. No.: 543-32205-00);
- Ultrasound cleaning unit—Elmasonic S40 (SKSONIC, Mörfelden-Walldorf, Germany, Cat. No.: 1004635);
- Vacuum device—Univapo 150 H & Unijet II Refrigerated Aspirator (Uniequip, Freital, Germany, Cat. No.: Univapo 150 H & Unijet II 20710);
- Vacuum sealer- Kitchenboss (Shenzhen Green Electrical Appliance Co., Ltd, Guangdong, China; Distributor: Amazon, Cat. No.: G200/silver);
- Vertical electrophoresis chamber-Mini-PROTEAN Tetra Cell (Bio-Rad Laboratories GmbH, Feldkirchen, Germany, Cat. No.: 1658000).

## 3. Procedure

### 3.1. Preparation and Performance of Hypoxic Cell Culture Experiment—Time for Completion: 5–72 h

1. Seed 1 × 10^5^ cells per well in degassed HEPES-buffered complete IMDM (with or without 100 µM CoCl_2_) in four wells of a 6-well plate. This first step ensures efficient cell adherence and adaptation. CoCl_2_ blocks any degradation of the hypoxia factor HIF-1α under normoxic conditions [15] when the hypoxic cell culture plate is opened later (see Section 3.2.2 and Section 3.2.3).
2. After 1 h of incubation of the cells under normoxic conditions at 37 °C and 7.5% CO_2_, add 0.3 g of oxygen absorber to the fifth well and a single AgelessEye indicator to the sixth well of the 6-well plate (Figure 1).
3. To allow gas exchange inside the 6-well plate, place two sterile 0.75 mm plastic spacers in a parallel alignment across the top and bottom three wells of the open cell culture plate and close it carefully with the corresponding lid (Figure 1).
4. To microscopically verify the hypoxic conditions and the cultured cells’ hypoxic state, supplement one of the cell-containing wells with Image-iT Green Hypoxia Reagent (final concentration of 5 μM). This fluorogenic compound is live cell-permeable and emits a green fluorescence in hypoxic environments.
5. Put the assembled cell culture plate into a vacuum bag. Insert the bag’s open end into the vacuum sealing machine and evacuate the air within the experimental unit to a low vacuum (Figure 1).
6. Place the assembled and evacuated hypoxic cell culture plate into a standard incubator and cultivate the cells for 5 h (or longer, e.g., 48 or 72 h) at 37 °C. As a control, a 6-well plate with corresponding cells (in the presence and absence of Image-iT Green Hypoxia Reagent) can be cultivated with complete IMDM in parallel for the same time under normoxic conditions. Further, for initial validation of the new hypoxic cell culture method, we additionally used a Whitley H35 hypoxystation (standard setting: ≤2% O_2_, 7.5% CO_2_, 90.5% N_2_) in control experiments.
7. After 30 min of incubation, control the oxygen concentration in the sealed culture plates visually by the color of the AgelessEye (it should be constant at 0.2–0.3%, corresponding to the range of oxygen values found in tissues and cells in vivo [1]).

### 3.2. Experimental Verification of Hypoxic Cell Culture Conditions by Immunofluorescence, Lactate Assay, and Immune Blot Analysis—Time for Completion Depends on Cultivation Period and Respective Downstream Analyses

#### 3.2.1. Detection of Low Oxygen Concentration in Living Cells by Hypoxia-Sensitive Fluorescence Dye after 5 h of Hypoxic Cultivation

1. After 5 h of hypoxic cultivation, remove the hypoxic cell culture device from the incubator, cut the vacuum bag at the sealed end, and remove the 6-well plate. When exposed to oxygen, the AgelessEye will turn from pink to purple and then blue (≥0.5%).
2. To distinguish living from dead cells, dispense 500 µL trypsin-EDTA solution into one of the culture vessels to completely cover the cells and place in the incubator at 37 °C for up to 5 min. This allows the cells to detach from the culture plate surface. This can be checked with an inverted microscope. When this is complete, all cells will be in suspension. Add complete IMDM (Iscove’s Modified Dulbecco’s Medium) containing FCS (Fetal Calf Serum) to the cell suspension to inhibit further tryptic activity. Mix one part of 0.4% trypan blue solution with one part cell suspension. Allow mixture to incubate for 3 min at room temperature and analyze by light microscopy. Do this for later analysis time points (e.g., for 48 and 72 h) as well.
3. Image the cells that were pretreated with Image-iT Green Hypoxia Reagent in the vessel using the fluorescence microscope with excitation/emission 488/520 nm (a standard FITC/GFP (Fluorescein Isothiocyanate/Green Fluorescent Protein) excitation/emission filter set is recommended). If necessary, the fluorescent cells can also be fixed with 2% formaldehyde. Fluorescence of the fixed cells lasts max. 24 h (afterward, it is hardly detectable). If cells are also grown on coverslips, they can be viewed at higher magnification with an appropriate fluorescence microscope (e.g., ApoTome microscope) after respective fixation and embedding in mounting medium.

#### 3.2.2. Measurement of Increased Glycolytic Lactate Production after 48 h of Hypoxic Cultivation

1. After 48 h of cultivation under norm- and hypoxia, monitor the cells for increased glycolysis via an L-lactate assay.
2. Remove the medium from the culture vessel by aspiration and wash cells with cold PBS.
3. Dispense 500 µL trypsin-EDTA (Ethylenediamine Tetraacetic Acid) solution into the culture vessel and place it in the incubator at 37 °C for up to 5 min to detach the cells from the culture plate surface. When this is complete, add complete IMDM containing FCS to the cell suspension to inhibit further tryptic activity.
4. Subsequently, determine the cell number by a Neubauer counting chamber.
5. Wash the detached/harvested cells by centrifugation at 200× *g* for 5 min and resuspend them in PBS at a concentration of 1 × 10^5^ cells/100 µL.
6. After further centrifugation of 300 µL cell suspension at 200× *g* for 5 min, resuspend the cell pellet in 200 µL of lactate assay buffer provided with the kit.
7. Homogenize the cells quickly by pipetting up and down ten times.
8. Centrifuge for 5 min at 4 °C and 1000× *g* in a cold centrifuge to remove insoluble material.
9. Deproteinize the resulting supernatants using a protein precipitating TCA (Tricarboxylic acid) kit according to the manufacturer’s protocol.
10. For the further steps of the L-lactate assay, follow the detailed protocol of the manufacturer. The colorimetric response of the assay was measured with an ELISA (Enzyme-Linked Immunosorbent Assay) reader at a wavelength of 450 nm.

#### 3.2.3. Detection of CoCl_2_-Stabilized HIF-1a Levels and PDK1 (Pyruvate Dehydrogenase Kinase 1) Induction in Cell Extracts after 72 h of Hypoxic Cultivation

1. For the preparation of cell lysates used for SDS-PAGE (Sodium Dodecyl Sulfate-Polyacrylamide Gel Electrophoresis)/immunoblot analysis, detach the cells with a trypsin-EDTA solution as described above.
2. Collect the detached cells in a centrifuge tube and centrifuge at 400× *g* for 5 min at 4 °C.
3. Resuspend the resulting cell pellet in 1 mL PBS centrifuge again at 400× *g* for 5 min at 4 °C. Subsequently, determine the cell number by a Neubauer counting chamber and resuspend the cell pellet in HIF-lysis buffer at a concentration of 1 × 10^5^ cells/100 µL.
4. Perform the cell lysis on ice for 30 min. Centrifuge the lysed cells at 1000× *g* and 4 °C for 30 min. Nuclei and cell debris will form a pellet so that the lysate supernatant can be transferred to a new reaction tube.
5. For denaturation and complexation with SDS, add one volume of 2× SDS sample buffer to the cell lysates and boil for 10 min in a heating block at 95 °C.
6. Load the polyacrylamide gel with the respective samples (containing an equal quantity of cell lysates (5 µL)) and a molecular weight marker (prestained protein ladder).
7. Run the gel at 100 V until the dye front migrates from the stacking into the running gel (15 min) and increase to 200 V until the dye front reaches the bottom of the gel (45 min).
8. Remove the gel from the apparatus, spacers, and glass plates and equilibrate it by soaking in transfer buffer for 2 min.
9. Prepare the nitrocellulose membrane by wetting it in transfer buffer for 30 s. Handle the membrane carefully, ideally with rounded tweezers, to avoid scratching or puncturing the surface.
10. Soak blotting filter papers and sponges in the transfer buffer for 5 min.
11. Starting on the side facing the cathode, sequentially assemble the following components: sponge, filter paper, gel, nitrocellulose membrane, filter paper, sponge. Gently remove any air bubbles with a roller or serological pipette. Bubbles between the gel and the membrane will inhibit the transfer of proteins to the membrane.
12. Place the completed transfer stack into a transfer cassette and perform wet transfer according to the manufacturer’s instructions for the blotting apparatus.
13. After transfer, rinse the membrane briefly in distilled water. Gently mark the position of the molecular weight ladder bands with a pencil for size detection. Using a scalpel, cut the membrane horizontally at the level of the 40 and 70 KDa markers of the prestained protein ladder.
14. Stain the membrane with Ponceau S for 30 s and then rinse briefly with distilled water to visualize protein bands and confirm that the protein transfer was successful. Wash away Ponceau S with several washes in PBS until the membrane is clear. Incubate membrane in PBS/0.1% (*v/v*) Tween 20/5% (*w/v*) milk powder solution for 1 h at room temperature with constant rocking.
15. Dilute the primary antibodies to the working concentration (anti-HIF-1α, 1:500; anti-PDK1, 1:500; anti-GAPDH, 1:1000) in PBS/0.1% Tween 20/10% (*v/v*) FCS.
16. Incubate the membrane in primary antibody solutions for 2 h at room temperature with gentle rocking (the upper part of the membrane with anti-HIF-1α, the middle part with anti-PDK1, and the lower part with anti-GAPDH).
17. Wash the membrane with PBS/0.1% (*v/v*) Tween 20 solution three times for 10 min each with gentle rocking.
18. Incubate the membrane with secondary antibody (goat anti-rabbit-HRP, 1:1000; goat anti-mouse-HRP, 1:1000) in PBS/0.1% (*v/v*) Tween 20 for 1 h at room temperature with gentle rocking (the upper/middle parts with anti-rabbit-HRP, and the lower part with anti-mouse-HRP).
19. Wash the membrane in PBS/0.1% (*v/v*) Tween 20 three times for 10 min each with gentle rocking.
20. Prepare the enhanced ECL (chemiluminescence) substrate just before use according to the manufacturer’s instructions.
21. According to the manufacturer’s suggestions, incubate the membrane in the substrate (typical incubation times are 10 s to 2 min).
22. Carefully remove the membrane from the detection reagent and sandwich it between layers of plastic (i.e., a sheet protector or plastic wrap).
23. Expose the membrane to autoradiography film in a dark room.
24. After the exposure is complete, place the film into the developer and wait until it is processed.
25. Once the film has been developed, overlay it back on your blot to mark the position of the protein ladder with a marker.

## 4. Expected Results

**Figure 2:**
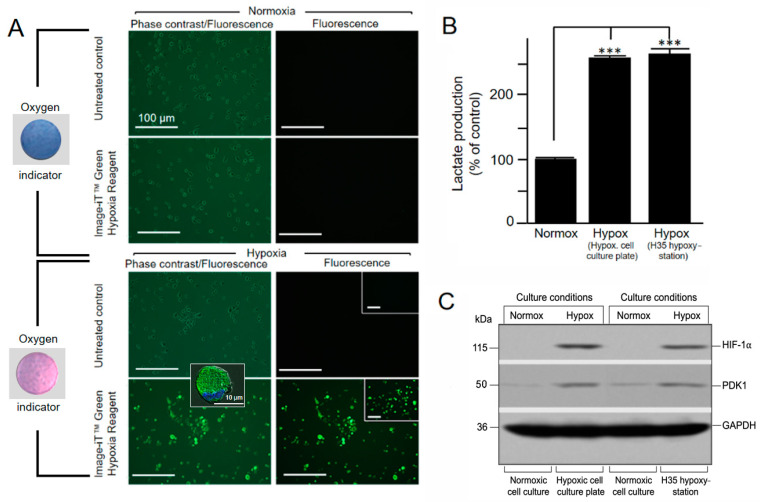
Cultivation of JAWS II cells under hypoxic cell culture conditions. (**A**) The microscopic images show the analysis of JAWS II cells after 5 h of cultivation under hypoxic cell culture conditions in a sealed hypoxic cell culture plate. Untreated and Image-iT Green Hypoxia-treated cells under norm- (top) and hypoxic (bottom) conditions are shown in phase contrast and under fluorescence (10× magnification). The corresponding color of the oxygen indicator is shown on the left. The lower left image insert shows a green fluorescent hypoxic cell taken with a Zeiss ApoTome fluorescence microscope using a 63× oil immersion objective (the cellular nucleus was costained with DAPI, blue). The inserts in the two lower right images show corresponding control and Image-iT Green Hypoxia-stained cells cultivated in a commercial hypoxystation. (**B**) JAWS II cells were cultured under norm-, and hypoxic conditions in either the hypoxic cell culture plate or a hypoxystation, as indicated, for 48 h after which cellular lactate was measured with an L-lactate assay kit (*** *p* < 0.001 versus controls; *n* = 3). (**C**) Immunoblot analysis of HIF-1α stability and PDK1 induction in JAWS II cells under norm- and hypoxic cell culture conditions after 72 h of cultivation in the hypoxic cell culture plate (second lane) and a commercial hypoxystation (fourth lane). GAPDH was included as a sample loading control.

During incubation of the sealed hypoxic cell culture plate in a standard incubator at 37 °C, the AgelessEye hypoxia sensor was used to visually monitor oxygen levels. Inspection at the beginning, middle, and end of the cell cultivation revealed that the sensor’s color remained light pink (0.2–0.3% O_2_) at all times, indicating a constant low oxygen concentration in the sealed culture plate. Moreover, trypan blue staining showed 91, 89, and 85% intact live cells after 5, 48, and 72 h of hypoxic incubation, respectively. This corresponds well to the survival rates obtained for cells cultured in the commercial hypoxystation. Microscopic analysis of the hypoxic cultivation conditions in the sealed hypoxic cell culture plates was performed for live JAWS II cells using the Image-iT Green Hypoxia Reagent, which sustains its fluorescence when cells/tissue return to normal oxygen levels. Figure 2A shows JAWS II cultured in the presence or absence of the Image-iT hypoxia detection reagent under hypoxic and standard normoxic conditions. Only the hypoxic cell cultures show the appearance of a bright green cellular fluorescence. The low-oxygen conditions generated in the hypoxic cell culture plate were verified by comparing the observed responses with control experiments using a commercial hypoxystation. As shown in Figure 2A, the fluorescence staining of cells treated with Image-iT in the hypoxic culture plate is comparable to those grown in the hypoxystation, suggesting that the two methods are highly comparable.

Increases in glucose consumption and the glycolytic rate during hypoxia lead to increased conversion of pyruvate to lactate and hence its accumulation in the cytoplasm. Thus, high lactate levels can be regarded as an indicator of cellular hypoxia. Indeed, as expected, there were increased levels of lactate in the cells cultured in the sealed hypoxic culture plate compared to the cells maintained under standard normoxic conditions (Figure 2B). Again, no difference was detected between cells cultured in the hypoxic culture plate or a commercial hypoxystation. Both cases showed a comparable increase in lactate levels. 

Finally, it is well known that CoCl_2_ enhances the stability of HIF-1α under normoxic conditions. In our experimental protocol, CoCl_2_ was added to hypoxic cell cultures to stabilize HIF-1α (and the cellular processes it controls) after opening the hypoxic cell culture plate for further downstream work/analyses under normal ambient air conditions. Immunoblot analyses showed a readily detectable HIF-1α-stabilization for the cells from the hypoxic culture plate, which was almost the same intensity as that found for hypoxic cells cultured in the hypoxystation (Figure 2C). No HIF-1a stabilization could be seen under normoxic cell culture conditions. Moreover, a comparable induction of the second hypoxia marker (PDK1), which inactivates the mitochondrial TCA cycle enzyme, pyruvate dehydrogenase (PDH) [16], could also be observed for the JAWS II cells cultured in the hypoxic cell culture plate and/or the commercial hypoxystation. 

Taken together, the sealed hypoxic cell culture plate has proven to be suitable for performing hypoxic cell culture experiments in standard incubators, and in particular, the additional use of CoCl_2_ allows further downstream analyses of HIF-1α related processes. Previously invented methods and procedures for performing hypoxia experiments in simple cell culture chambers [17,18,19] have the additional expense and complexity of gas mix supply/discharge. This is not necessary for the sealed six-well plate system presented here and greatly simplifies the entire experimental procedure.

Although the presented method has experimental limitations (e.g., a static hypoxic O_2_ concentration) and cannot fully replace all the capabilities of a commercial hypoxystation, it opens new possibilities for the simple and inexpensive experimental application of hypoxic cell culture conditions in pilot experiments. A further application of this approach is cell culture work performed under higher biological safety levels (such as BSL3 and 4) where sophisticated hypoxia chambers are less likely to be available, and where their use might not be ideal for a variety of practical and safety reasons.

## 5. Reagents Setup

Blotting buffer: 25 mM Tris–HCl (pH 7.5), 190 mM glycine. Store at room temperature.

Complete IMDM with 25 mM HEPES and 20% FCS: Add 2.95 g of HEPES and 100 mL of FCS to 400 mL of IMDM (supplemented with penicillin–streptomycin (100 U/mL)), amphotericin B (2.5 µg/mL), and GM-CSF (5 ng/mL mouse GM-CSF). Adjust to pH 7.4 with HCl.

Electrophoresis buffer: 25 mM Tris–HCl (pH 7.5), 190 mM glycine, 10% (*v/v*) SDS. Store at room temperature.

HIF-Lysis buffer: 6.65 M urea, 10 mM Tris (pH 6.8), 1% SDS, 10% glycerin, and one tablet of cOmplete protease inhibitor per 50 mL of lysis buffer.

Immunoblot blocking buffer: 1× PBS, 0.1% (*v/v*) Tween 20, 5% (*w/v*) milk powder. Prepare freshly (short-term storage on ice).

Immunoblot washing buffer: 1× PBS, 0.3% (*v/v*) Tween 20. Store at room temperature.

Loading buffer: 10 mM Tris–HCl (pH 6.8); 6.6 M urea; 1% (*v/v*) SDS; 10% (*v/v*) glycerin; 0.25% (*w/v*) bromophenol blue; 7% (*v/v*) 2-mercaptoethanol, and one tablet of cOmplete protease inhibitor per 50 mL of loading buffer.

PBS (phosphate-buffered saline): For 1000 mL of 1× PBS, start with 800 mL of distilled water. Then, add 8 g of NaCl, 0.2 g of KCl, 1.44 g of Na_2_HPO_4,_ and 0.24 g of KH_2_PO_4_. Adjust the pH to 7.4 with HCl. Add distilled water to a total volume of 1000 mL (final concentration of 137 mM NaCl, 10 mM phosphate, 2.7 mM KCl). Sterilize the solution by autoclaving for 30 min and degas the buffer by sonication (in an ultrasonic bath for 45 min at room temperature and by applying a vacuum for 60 min. Store the PBS buffer at room temperature.

Ponceau S solution: 0.5% (*w/v*) Ponceau S dissolved in 1% (*v/v*) acetic acid.

Primary and secondary antibody incubation buffer: 1× PBS, 0.1% (*v/v*) Tween 20, 10% (*v/v*) fetal calf serum (FCS). Prepare fresh (short-term storage on ice until use).

Separating gel (10%): Mix in the following order: H_2_O: 4.1 mL; acrylamide/bis (30% 37.5:1): 3.3 mL; Tris–HCl (1.5 M, pH 8.8): 2.5 mL; 10% (*v/v*) SDS: 100 µL; TEMED: 10 µL; 10% (*v/v*) ammonium persulfate (APS): 32 µL.

Stacking gel (4%): Mix in the following order: H_2_O: 6.1 mL; acrylamide/bis (30%, 37.5:1): 1.3 l; Tris–HCl (0.5 M, pH 6.8): 2.5 mL; 10% (*v/v*) SDS: 100 µL; TEMED: 10 µL; 10% (*v/v*) APS: 100 µL.

Transfer buffer: 25 mM Tris–HCl (pH 7.5), 190 mM glycine. Store at room temperature.

Trypan Blue Solution: Dissolve 0.4 g of Trypan Blue in 80 mL of 1× PBS and bring to a slow boil. Cool to room temperature and add PBS to a final volume of 100 mL. Store at room temperature.

## Data Availability

All data generated or analyzed during this study are included in this published article.
